# Personalized Hypertension Management Using Patient-Generated Health Data Integrated With Electronic Health Records (EMPOWER-H): Six-Month Pre-Post Study

**DOI:** 10.2196/jmir.7831

**Published:** 2017-09-19

**Authors:** Nan Lv, Lan Xiao, Martha L Simmons, Lisa G Rosas, Albert Chan, Martin Entwistle

**Affiliations:** ^1^ Palo Alto Medical Foundation Research Institute Palo Alto, CA United States; ^2^ Office of Patient Experience Sutter Health Mountain View, CA United States; ^3^ Department of Medicine Stanford University School of Medicine Palo Alto, CA United States; ^4^ Ares Health Solutions Pasadena, CA United States

**Keywords:** electronic health records, disease management, hypertension, patient participation, blood pressure, patient-centered care, home blood pressure monitoring

## Abstract

**Background:**

EMPOWER-H (Engaging and Motivating Patients Online With Enhanced Resources-Hypertension) is a personalized-care model facilitating engagement in hypertension self-management utilizing an interactive Web-based disease management system integrated with the electronic health record. The model is designed to support timely patient-provider interaction by incorporating decision support technology to individualize care and provide personalized feedback for patients with chronic disease. Central to this process were patient-generated health data, including blood pressure (BP), weight, and lifestyle behaviors, which were uploaded using a smartphone.

**Objective:**

The aim of this study was to evaluate the program among patients within primary care already under management for hypertension and with uncontrolled BP.

**Methods:**

Using a 6-month pre-post design, outcome measures included office-measured and home-monitored BP, office-measured weight, intervention contacts, diet, physical activity, smoking, knowledge, and health-related quality of life.

**Results:**

At 6 months, 55.9% of participants (N=149) achieved office BP goals (<140/90 mm Hg; *P*<.001) and 86.0% achieved clinically meaningful reduction in office BP (reduction in systolic BP [SBP] ≥5 mm Hg or diastolic BP [DBP] ≥3 mm Hg). At baseline, 25.2% of participants met home BP goals (<135/85 mm Hg), and this percentage significantly increased to 71.4% (*P*<.001) at 6 months. EMPOWER-H also significantly reduced both office and home SBP and DBP, decreased office-measured weight and consumption of high-salt and high-fat foods (all *P*<.005), and increased intake of fruit and vegetables, minutes of aerobic exercise, and hypertension knowledge (all *P*<.05). Patients with higher home BP upload frequencies had significantly higher odds of achieving home BP goals. Patients receiving more total intervention, behavioral, pharmaceutical contacts had significantly lower odds of achieving home BP goals but higher improvements in office BP (all *P*<.05).

**Conclusions:**

EMPOWER-H significantly improved participants’ office-measured and home-monitored BP, weight, and lifestyle behaviors, suggesting that technologically enabled BP home-monitoring, with structured use of patient-generated health data and a personalized care-plan facilitating patient engagement, can support effective clinical management. The experience gained in this study provides support for the feasibility and value of using carefully managed patient-generated health data in the day-to-day clinical management of patients with chronic conditions. A large-scale, real-world study to evaluate sustained effectiveness, cost-effectiveness, and scalability is warranted.

## Introduction

Hypertension is a major risk factor for cardiovascular disease (CVD), with an estimated annual cost of US $46 billion in the United States [1,2]. In national data, 29% of American adults have been diagnosed with hypertension and among those, only 52% have adequately controlled blood pressure (BP) under the recommended level of 140/90 mm Hg [3]. An additional 28% of Americans have prehypertension, a precursor of hypertension characterized by BP of 120/80 mm Hg to 139/89 mm Hg [4]. Furthermore, the problem is growing; it is estimated that 41% of American adults will have hypertension by 2030, with an estimated annual cost of US $274 billion [1]. Although common and costly, hypertension is preventable and modifiable through promotion of a healthy lifestyle, such as weight loss, healthy diet, and physical activity, or improving medication adherence.

Health policies have begun to shift away from episodic management of individual patients toward managing and paying for ongoing health care services that drive engagement and achieve valuable outcomes for groups of patients [5]. Population health management, the proactive application of strategies and interventions to defined groups of patients in an effort to improve health efficiently and at the lowest necessary cost [6], has been shown to be effective and cost-effective for conditions such as hypertension [7-9]. Population health management to prevent and control hypertension requires scalable and sustainable lifestyle interventions. Pragmatic technology-assisted approaches offered by the existing health care infrastructure may facilitate patient self-management and increase the potential for widespread reach and adoption, resulting in improved long-term effectiveness and a shift toward a population-based management model. Growing evidence [10,11] suggests that new approaches including technology-assisted clinical tools can both increase access and decrease cost for primary care–based hypertension prevention and management programs that traditionally place a heavy burden on personnel and resources. For example, technologies such as the Internet-based home BP telemonitoring can increase patient engagement and alert out-of-limit BP readings, which is a proven strategy for improved BP control [12-14]. Such technology-assisted programs have the potential to assist patients in self-managing their chronic conditions, including support for lifestyle changes, thus reducing reliance on health care personnel. Whereas evidence suggests that technology-assisted interventions in health care settings for hypertension prevention and control are potentially scalable and cost-effective, best practices remain unknown.

The Engaging and Motivating Patients Online With Enhanced Resources (EMPOWER) program is an innovative care delivery model for chronic disease management utilizing an interactive Web-based system integrated with the electronic health record (EHR). The model is designed to support timely patient-provider interaction by incorporating decision support technology to individualize care. The program also supports patient self-management and engagement by providing real-time feedback on progress against clinical goals to facilitate care-plan engagement. The model is designed to be generalizable to any chronic condition where behavior change has a direct impact on health outcomes. In this pilot study, we sought to evaluate the EMPOWER-Hypertension (EMPOWER-H) program among patients with uncontrolled BP in the context of primary care, using data from home BP monitoring as a major input to clinical decision making.

## Methods

We evaluated the EMPOWER-H intervention using a pre-post design. An institutional review board of Palo Alto Medical Foundation (PAMF), an affiliate of Sutter Health, approved the study. All participants provided written informed consent.

### Study Participants

**Figure 1:**
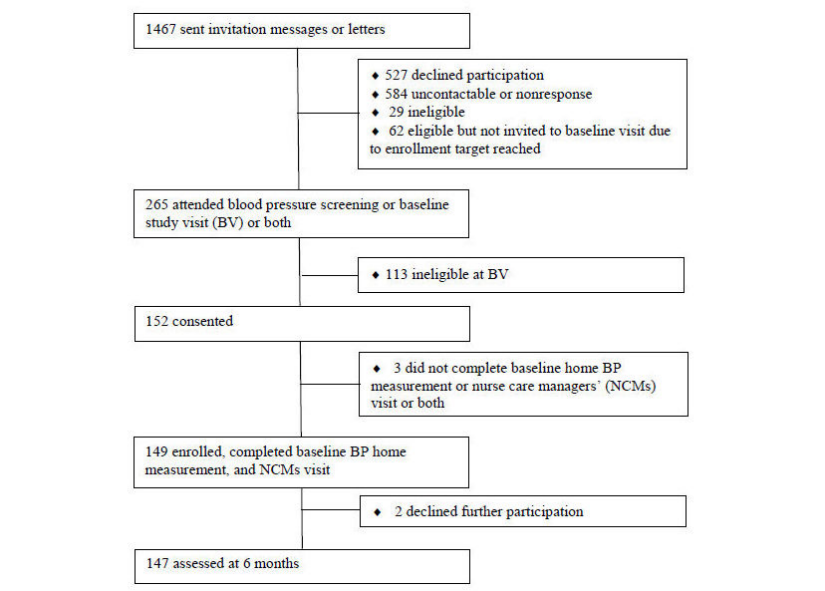
Consolidated Standards of Reporting Trials (CONSORT) chart.

Participants were recruited (March 2012 to June 2012) from two clinical sites at PAMF, a large ambulatory health care system. Patients were eligible to participate in the pilot study if they were in the age range of 35 to 75 years, had uncontrolled BP, and had been treated within PAMF during the previous 6 months. Participants were considered to have uncontrolled BP if (1) the average of 2 BP readings taken within 6 months of the study start date and separated by at least 2 weeks was 140 mm Hg to 175 mm Hg for SBP or 90 mm Hg to 110 mm Hg for DBP or both and (2) BP was within this same range when taken by the research staff during an in-person baseline visit. Exclusion criteria included having serious medical conditions (eg, diabetes, renal failure type III or IV, coronary artery disease, or stroke), requiring management of psychiatric issues, being under primary care management for a diagnosis of hypertension for less than 6 months, participating in other research projects, or having special life circumstances (eg, pregnancy, planned relocation, past or planned gastric bypass, or having no insurance). Of the 1467 patients contacted for recruitment, 527 patients declined participation, 142 were ineligible, 584 were not contactable or nonresponsive, 62 were interested but not enrolled as the study reached full enrollment before their initial study visit, and 3 consented but did not complete baseline home BP measurement or nurse care managers’ (NCMs) visit (first in-person intervention visit). This process yielded the target enrollment of 149 eligible and consenting participants (Figure 1).

### Recruitment and Screening Process

Recruitment began by querying the EHR to identify patients who met the inclusion criteria. Upon identification, a clinical study coordinator conducted chart reviews to verify BP readings during the previous 6 months, as well as other exclusion criteria. A list of potentially eligible patients was provided to primary care physicians (PCPs) for final approval. After approval, PCPs contacted their eligible patients either via a secure Web-based patient portal (My Health Online) message, or by letter and encouraged them to participate in the study. The Web-based patient portal is an integral part of the PAMF EHR. Patients can log in to message their medical providers, view lab results, and receive information about appointments. The initial communication included instructions on accessing the study enrollment site where the patient could read a description of the study, provide consent online, complete a self-screening questionnaire (eg, age, and pregnancy) and, if eligible, complete a baseline questionnaire.

For patients who were eligible per the initial Web-based screening, trained research staff arranged an in-person baseline visit where written consent was attained, and BP was measured in the office using the patient’s home BP monitor according to standardized protocols [15] to confirm that the patient met the BP inclusion criteria.

### Intervention

EMPOWER-H was a 6-month intervention delivered by a care team, including 2 NCMs, a registered dietitian (RD), and a pharmacist for consultation. The intervention was based on theoretical constructs such as perceived severity of a health threat; relevant values; self-efficacy; perceived barriers to action, as described in the theory of planned behavior [16]; health belief model [17]; and social cognitive theory [18]. It used an enhanced version of the Web-based disease management system as deployed to support EMPOWER-Diabetes (EMPOWER-D) [19], a precursor of EMPOWER-H. EMPOWER-H included (1) a wireless BP monitor that transmitted home BP readings to PAMF’s EHR and the EMPOWER system; (2) a smartphone (Apple iPhone 3) with 2 study apps (described below); (3) a comprehensive dashboard of the status of a patient’s personalized action plan, treatment goals, and self-monitoring data, available directly from within PAMF’s Web-based patient portal (My Health Online); (4) a pedometer for monitoring steps; (5) Web-based messaging system for communicating between patients and members of the care team; (6) NCMs assisted by RD for nutrition and weight management and pharmacist to provide consultation and make medication changes; and (7) patient-specific text and video educational nuggets (Multimedia Appendix 1). On the smartphone, the Numera app allowed wireless transmission of the home BP data to the smartphone and then to the EHR and EMPOWER system. The EMPOWER-H app displayed the patient-generated home data, allowed visualization and tracking of personal goals, and provided access to educational nuggets.

At the baseline visit, research staff provided eligible patients with their study tools as described above, including the BP monitor with appropriate sized cuff, a pedometer, and a smartphone, along with instructions for use. Research staff also introduced patients to their personal dashboard and provided educational handouts and actions to take in case of very high or very low BP measurements. Patients were instructed to measure and upload BP readings in the morning and evening at regular times or events (eg, before breakfast or dinner) for at least 3 days a week over the following 7 days (baseline home BP monitoring) and to continue the same pattern of uploading during the entire 6-month intervention. They were also encouraged to use their pedometer and upload daily step count. A scale that allowed wireless upload of weight was provided to selected patients at a later visit, based on clinical indications determined by an RD.

Participants had a first in-person visit with an NCM as soon as possible after the 7-day baseline home BP collection period. This visit included the following activities: (1) addressing participants’ questions; (2) providing education about cardiovascular risks; (3) reviewing how to use study tools; (4) setting 2 to 3 small attainable goals utilizing motivational interviewing techniques; (5) visiting the Web-based dashboard, entering the personalized goals, and showing patients how to enter and view their data; and (6) developing a personalized BP management plan (eg, frequency of contacts for check-ins, goal-setting, and data upload) informed by the 7-day baseline home BP. Home BP goals were set at American Heart Association’s (AHA) recommended levels of <135 mm Hg for SBP and <85 mm Hg for DBP [20]. The NCM encouraged all patients to have a separate visit with an RD to discuss diet history, current dietary habits, and recommended changes. The vast majority (95.3%) of patients had at least one contact with an RD.

The NCM contacted each patient via EHR secure messaging or telephone or both as soon as possible 1 week after the first visit to assess engagement, challenges with both the technology and clinical management, as well as to check BP control and modify the BP management plan if needed.

During the 6-month intervention, patients were monitored daily using a provider dashboard which used both a 14-day average of home BP data and frequency of BP uploading (as a measure of engagement) to prioritize patients into high-risk (red), medium-risk (yellow), or low-risk (green) category (Multimedia Appendix 2). The dashboard also provided alerts if individual home BP measurements were at a critical level (SBP ≤90 or ≥160; DBP ≤55 or ≥100). NCMs who worked with an RD and pharmacist strictly followed the hypertension clinical management protocol that was developed for registered nurse (RN) management of hypertension based on the 7th report of the Joint National Committee on Prevention, Detection, Evaluation, and Treatment of High Blood Pressure [2] and approved by PAMF nursing governance to provide continuous patient support. The protocol provides narrowly defined parameters within which an NCM may make adjustments to medical and therapeutic management, including limits on how frequently management adjustments may be made. Situations that fall outside the defined areas for modifications to management must be referred to the treating physician, a PCP, or a specialist for determination of action. When further assistance with medication adjustment was needed, the pharmacist reviewed charts and consulted with an NCM.

An NCM or RD had a library of Web-based education handouts and feedback message nuggets (texts, links to Web pages, or short videos) that could be sent via secure messaging on the Web-based patient portal throughout the intervention. NCMs and RD primarily used secure messaging to provide regular pertinent electronic messages and timely feedback about participants’ clinical variables (eg, home BP readings, medication doses, weight, and steps). In addition to these Web-based learning opportunities, participants were invited to participate in other engagement activities throughout the intervention, including healthy shopping tours and cooking classes at a grocery store, educational and interactive webinars on behavioral change, a pedometer challenge, and a healthy recipe challenge.

### Outcome Measures

The 149 enrolled participants were assessed at baseline and 147 (98.7%) were assessed at 6 months (Figure 1). All outcome assessors were trained to perform the measurements per standardized protocols and procedures (Multimedia Appendix 3).

We assessed the effectiveness of the intervention using BP measurements taken in the office by trained research staff (office BP) and taken at home by the patient (home BP) as part of self-monitoring for the intervention. Office BP was measured by trained research staff according to standardized protocols [15]. The primary outcome for this study was the percentage of participants achieving office BP goals (<140 mm Hg for SBP and <90 mm Hg for DBP) at 6 months as measured in controlled circumstances. Secondary BP outcomes included change in office BP, percentage of participants achieving clinically meaningful reductions in office BP (a drop in SBP of ≥5 mm Hg or a drop in DBP of ≥3 mm Hg) [21-24], change in home BP measurements, and percentage of participants achieving home BP goals (<135 mm Hg for SBP and <85 mm Hg for DBP) at 6 months. Baseline home BP was calculated as an average of BP self-monitored during the 7 days after baseline, and 6-month home BP was an average of BP self-monitored during the 7 days before the 6-month visit.

Other secondary outcomes included number of participants meeting weekly home BP monitoring frequency target (upload twice a day and 3 days in a week), body mass index (BMI), weight, dietary intake, physical activity, smoking status, hypertension knowledge, as well as health-related quality of life (HRQoL). Home-monitored BP data were extracted from the EHR that pulled data from the Numera database. Indicators of dietary intake included frequency of consuming fruit and vegetables, high-salt food, and high-fat food measured by scales adapted from the validated Block food screeners [25,26]. Physical activity was measured by the Stanford Exercise Behavior Scale [27]. Hypertension knowledge was measured using a 13-item knowledge questionnaire. HRQoL was measured by the Veterans RAND 12-Item Health Survey (VR-12) [28-31]. The VR-12 was developed from the Veterans RAND 36-Item Health Survey that was developed and modified from the original RAND version of the 36-item Health Survey version 1.0 (also known as the “MOS SF-36”).

Indicators of engagement in the intervention included total number of times patients uploaded BP measurement, pedometer step data, weight, stress (ie, stress level on a 5-point scale), and medication (ie, whether medication was taken as prescribed) to the Numera and EMPOWER-H apps. Other indicators of intervention engagement included participation in 6 additional activities designed to support education and motivation (ie, pedometer and recipe challenges, 2 cooking classes, and 2 learning webinars).

In addition, we tracked the number of intervention contacts (ie, total or patient-initiated intervention visits, My Health Online messages, phone messages, or phone calls on behavioral, pharmaceutical, laboratory, or technical issues).

Participant activation was measured by the 13-item Patient Activation Measure (PAM) assessing patient’s knowledge, skill, and confidence for self-management [32,33]. PAM score has a range of 0 to 100, with higher score indicating higher patient activation.

### Statistical Analyses

To examine our primary outcome, we used a one sample proportion test to compare the percentage of participants achieving office BP goals (<140 mm Hg for SBP and <90 mm Hg for DBP) at 6 months with the 0% who were meeting goals at baseline. As supplementary analyses, we compared the percentage of participants achieving office BP goals at 6 months with the percentage of patients who achieved normal BP with usual care alone in the same clinics during a similar period (30%). Frequencies and percentages were calculated for categorical variables, and means and standard deviations (SD) were calculated for continuous variables. Study outcomes at baseline and 6 months were compared using paired *t* tests for continuous outcomes and McNemar tests on paired proportions for categorical outcomes. McNemar exact tests were performed for the outcomes with small counts within certain categories (eg, 20% of cells with expected frequencies <5). We used mixed model growth curve analysis for all available home-monitored BP during the study period and plotted the results according to time. We analyzed SBP and DBP as an outcome in two separate models; each included random intercept and random and fixed effects of slope and quadratic term of days from start. The quadratic term of days was removed in the final model for both home-monitored SBP and DBP because it was not significant. We used logistic regression and regression models to examine the bivariate associations of indicators of intervention engagement with the categorical and continuous BP outcomes, respectively.

All analyses were conducted in Statistical Analysis System (SAS) version 9.3 (SAS Institute Inc). Statistical significance was set at *P*<.05 (two-tailed). We powered this study on the primary outcome of percentage of participants achieving office BP goals (<140 mm Hg for SBP and <90 mm Hg for DBP) at 6 months. A sample of 149 had 80% power to detect at least 40% of participants achieving office BP goals at 6 months at alpha=.05 (two-sided), assuming at least 90% retention at 6 months based on our prior experiences. We based our estimates on the 30% transition rate from abnormal BP to normal BP without intervention using patient data from our EHR assessed from 2009 to 2010.

## Results

### Baseline Characteristics

Participants had a mean (SD) age of 62.2 (9.5) years, 51% were female, 73% were married or lived with partner, and 76% were non-Hispanic white (Table 1). Most participants completed at least some college, had an annual family income of >US $75,000, were full-time employees or retired, and were never or former smokers. At baseline, their mean (SD) for BMI was 28.7 (6.2) kg/m^2^, SBP was 149.8 (9.8) mm Hg, DBP was 91.0 (8.0) mm Hg, and PAM score was 43.1 (6.1).

**Table 1:** Baseline characteristics of the study participants.

Characteristic		All participants (N=149)
Age in years, mean (SD^a^)		62.2 (9.5)
Female, n (%)		76 (51.0)
**Marital status, n (%), n=147**		
	Married or partner or domestic partnership	107 (72.8)
	Divorced or separated or widowed	25 (17.0)
	Single	15 (10.2)
**Race or ethnicity, n (%), n=146**		
	Non-Hispanic white	111 (76.0)
	Non-Hispanic black	9 (6.2)
	Hispanic	4 (2.7)
	Asian	18 (12.3)
	Other	4 (2.7)
**Education, n (%), n=147**		
	High school or GED^b^ or less	5 (3.4)
	College, 1-3 years	28 (19.1)
	College, 4 years or more	44 (29.9)
	Postgraduate	70 (47.6)
**Annual family income in US dollars, n (%), n=111**		
	$35,000-<$75,000	19 (17.1)
	$75,000-<$100,000	28 (25.2)
	$100,000-<$125,000	18 (16.2)
	$125,000-<$150,000	10 (9.1)
	$150,000+	36 (32.4)
**Employment status, n (%), n=147**		
	Full-time	67 (45.6)
	Part-time	10 (6.8)
	Homemaker	8 (5.5)
	Retired	49 (33.3)
	Unemployed or disabled or something else	13 (8.8)
**Smoking status, n (%)**		
	Never smoked	101 (67.8)
	Current smoker	8 (5.4)
	Former smoker	40 (26.9)
Body mass index, kg/m^2^, mean (SD), n=140		28.7 (6.2)
Systolic blood pressure, mm Hg, mean (SD), n=145		149.8 (9.8)
Diastolic blood pressure, mm Hg, mean (SD), n=145		91.0 (8.0)
Patient activation measure score^c^, mean (SD)		43.1 (6.1)

^a^SD: standard deviation.

^b^GED: general education diploma.

^c^13-item Patient Activation Measure (PAM) assessing patient’s knowledge, skill, and confidence for self-management has a range of 0 to 100. Higher score indicates higher patient activation.

**Table 2:** Changes in office-measured blood pressure (BP), home-monitored BP, and body mass index at 6 months.

Outcome variable		n^a^	Baseline	6 months	Change	*P* value
**Office-measured BP**^b^		149, 143, 143				
	Participants achieving BP goals, %^e^		0	55.9	55.9	<.001
	Systolic BP, mm Hg, mean (SD^d^)		149.8 (9.8)	134.4 (14.0)	−15.2 (15.3)	<.001
	Diastolic BP, mm Hg, mean (SD)		91.0 (8.0)	84.5 (8.6)	−6.4 (7.5)	<.001
**Home-monitored BP**		147, 133, 132				
	Participants achieving BP goals, %^c^		25.2	71.4	46.2	<.001
	Systolic BP, mm Hg, mean (SD)		138.4 (10.6)	126.7 (9.8)	−11.7 (11.5)	<.001
	Diastolic BP, mm Hg, mean (SD)		84.5 (7.8)	78.6 (6.9)	−6.1 (6.5)	<.001
Body mass index, kg/m^2^, mean (SD)		140, 136, 132	28.7 (6.2)	28.3 (6.0)	−0.4 (1.5)	.01
Weight, kg, mean (SD)		142, 138, 134	84.0 (21.7)	82.0 (20.7)	1.2 (4.5)	.002

^a^n is provided for baseline, 6 months, and change in order.

^b^BP: blood pressure.

^c^Office BP goals are <140 mm Hg for systolic and <90 mm Hg for diastolic.

^d^SD: standard deviation.

^e^Home BP goals are <135 mm Hg for systolic and <85 mm Hg for diastolic.

### Changes in Clinical Outcomes

Table 2 shows that 55.9% of the participants achieved office BP goals (<140 mm Hg for SBP and <90 mm Hg for DBP) at 6 months (*P*<.001). Additionally, this was also significantly higher than 30%, which was achieved with usual care alone based on 2009-2010 patient data in EHR (*P*<.001). Paired *t* test results show that mean (SD) systolic office BP was significantly reduced from 149.8 (9.8) to 134.4 (14.0) mm Hg and mean (SD) diastolic office BP from 91.0 (8.0) to 84.5 (8.6) mm Hg (both *P*<.001). At 6 months, 86% of participants achieved clinically meaningful reduction in office BP (reduction in SBP ≥5 mm Hg or reduction in SBP ≥3 mm Hg).

**Figure 2:**
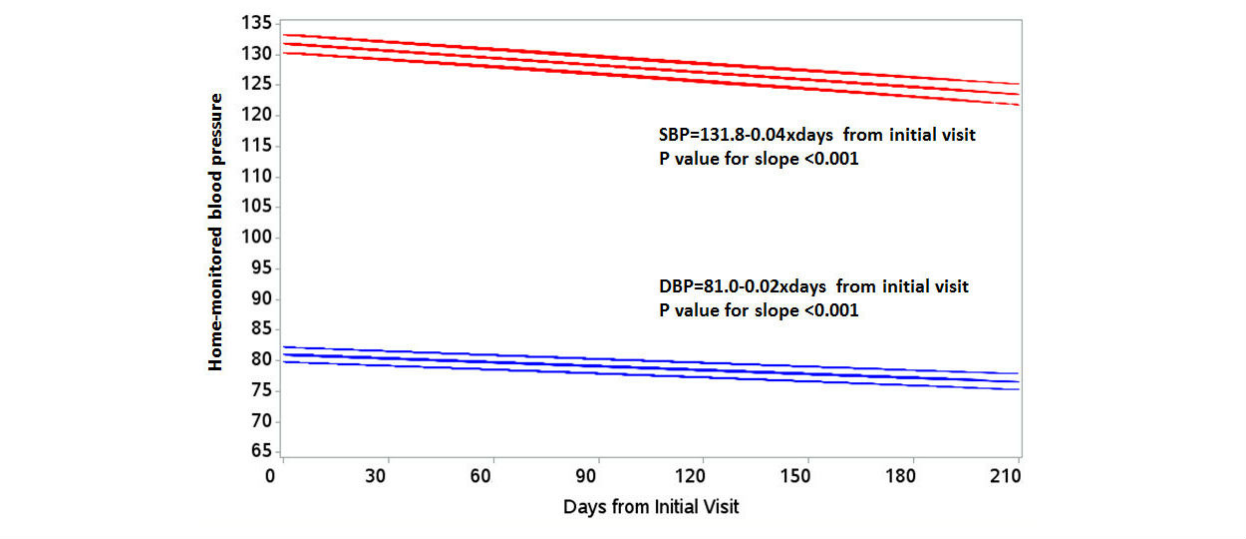
Predicted means and 95% CIs resulting from the random coefficient regression model of home-monitored blood pressures (N=149).

Home-monitored BP measurements show that percent of participants achieving home BP goals (<135 mm Hg for SBP and <85 mm Hg for DBP) significantly increased from 25.2% to 71.4% (*P*<.001), with a mean (SD) reduction of 11.7 (11.5) mm Hg for systolic and 6.1 (6.5) mm Hg for diastolic home BP (both *P*<.001; Table 2). Figure 2 shows significant linear downward trends in both home-monitored SBP and DBP (both *P*<.001). Of 132 participants with both baseline and 6-month home BP measurements, 27 (20%) started and stayed within the home BP goal at 6 months, 67 (51%) were not at goal at baseline and were meeting goal at 6 months, 6 (5%) started within goal but were above goal at 6 months, and 32 (24%) started and stayed outside goal at 6 months (data not shown). Comparison between office and home BP shows that change in office and home BP at 6 months was significantly correlated (*P*<.001 for SBP and *P*=.005 for DBP; data not shown).

Compared with baseline, participants’ BMI and body weight were significantly reduced (*P*=.01 for BMI and *P*=.002 for body weight; Table 2). Of the 149 participants, 121 (81.2%) received a wireless weight scale. There was no significant correlation between number of weight uploads and weight loss (*P*=.49)

### Changes in Behavioral Outcomes

Participants significantly increased consumption frequency of fruit and vegetables (*P*=.01) and reduced consumption frequency of high-salt and high-fat foods (both *P*<.001; Table 3). Minutes of aerobic exercise per week significantly increased (*P*=.03), whereas minutes of stretching or strengthening remained the same (*P*=.91). Participants’ hypertension knowledge also significantly improved (*P*<.001); however, smoking status and HRQoL measured by VR-12 remained unchanged (*P*>.05 for all; Table 3).

### Correlation of Intervention Engagement With Changes in Office-Measured and Home-Monitored Blood Pressure (BP)

Almost half (44.3%) of participants did at least one of the 6 additional activities designed to support sustained program engagement (pedometer and recipe challenges, 2 cooking classes, and 2 learning webinars). None of the intervention engagement measures (ie, number of home BP, pedometer data, weight, stress, and medication uploads, challenges, optional events, and intervention contacts) were associated with achieving office BP goals or clinically meaningful office BP improvement at 6 months (Table 4). However, a higher number of total interventions, behavioral, pharmaceutical, and total patient-initiated intervention contacts were significantly associated with higher improvements in values of systolic or diastolic office BP or both.

**Table 3:** Changes in behavioral outcomes, knowledge, and quality of life at 6 months.

Outcome variable		n^a^	Baseline	6 months	Change	*P* value
**Diet, times/week, mean (SD**^b^**)**		147, 140, 138				
	Fruit and vegetables^c^		25.5 (10.5)	27.9 (11.3)	2.3 (10.8)	.01
	High-salt food^d^		8.4 (5.1)	6.5 (3.8)	−1.8 (4.5)	<.001
	High-fat food^e^		15.9 (8.9)	12.5 (7.0)	−3.3 (6.3)	<.001
**Physical activity, minutes/week, mean (SD)**		147, 140, 138				
	Stretching or strengthening		51.7 (60.3)	53.0 (58.9)	−0.5 (55.7)	.91
	Aerobic exercise		178.6 (132.4)	206.4 (126.2)	25.7 (133.1)	.03
**Smoking status, %**		149, 139, 139				.15
	Never smoker		67.8	66.9		
	Current smoker		5.4	2.9	N/A	
	Former smoker		26.9	30.2		
Hypertension knowledge, mean (SD)^f^		147,140,138	11.3 (1.6)	12.3 (1.0)	1.0 (1.4)	<.001
VR-12, physical component score, mean (SD)^g^		147,140,138	49.7 (8.0)	50.2 (7.8)	0.5 (7.1)	.39
VR-12, mental component score, mean (SD)^g^		147,140,138	53.1 (7.2)	53.5 (8.3)	0.1 (8.6)	.90

^a^n is provided for baseline, 6 months, and change in order.

^b^SD: standard deviation.

^c^Sum of eating frequency (0.5=less than 1/week or never, 1=once a week, 2.5=2 to 3 times a week, 5=4 to 6 times a week, 7=once a day, and 14=2 or more times a day) for 7 fruit and vegetable subgroups (fruit juice; fresh, canned, or frozen fruit; vegetable juice; green salad; potatoes; vegetable soup or stew with vegetables; and any other vegetables, including string beans, peas, corn, broccoli, or any other kind).

^d^Sum of eating frequency (0.5=less than 1/week or never, 1=once a week, 2.5=2 to 3 times a week, 5=4 to 6 times a week, 7=once a day, and 14=2 or more times a day) for 6 high-salt food consuming and using behaviors (restaurant food; packaged snack foods such as chips, pretzels, popcorn, salted nuts; canned soups, canned vegetables, or frozen meals or TV dinners; cured or salted meats; add salt to your food at the table; add any of the following to your food when preparing meals or eating out: salt, mustard, pickles, relish, soy sauce, ketchup, meat tenderizer, or MSG).

^e^Sum of eating frequency (0.125=less than 1 time per month or never, 0.375=2 to 3 times a month, 1.5=1 to 2 times a week, 3.5=3 to 4 times a week, and 6=5+ times a week) for 17 high-fat foods or food subgroups (hamburgers, ground beef, meat burritos, tacos, enchiladas; pork chops, beef; fried chicken; hot dogs or Polish or Italian sausage, organ meats; cold cuts, lunch meats, ham; bacon or breakfast sausage; salad dressing; margarine, butter, lard or mayo spread on bread or potatoes; margarine, butter, lard or oil in cooking; eggs; pizza; cheese, cheese spread; whole milk or chocolate milk; French fries or fried potatoes; corn chips, potato chips, popcorn, crackers, peanuts; doughnuts, pastries, cake, cookies, pan dulce; ice cream).

^f^Hypertension knowledge was measured using a 13-item knowledge questionnaire. Higher score indicates better knowledge.

^h^Physical and mental component scores are calculated to report an overall measure of physical and mental functioning that is comparable among the surveys. These summary scales have been normalized in the US population (value=50). The higher score indicates better self-reported health-related quality of life.

**Table 4:** Bivariate associations of office-measured blood pressure (BP) with number of home BP, pedometer, weight, stress, and medication uploads and number of intervention contacts (n=143).

Intervention engagement measures		Achieving BP goals^a^ OR^b^ (95% CI)	Achieving clinically meaningful BP^c^ improvement^d^ OR (95% CI)	SBP^e^ change^f^ coefficient (95% CI)	DBP^g^ change^f^ coefficient (95% CI)
Number of weeks meeting home BP monitoring frequency target		1.04 (1.00-1.08)	0.98 (0.93-1.04)	−0.16 (−0.47 to 0.16)	0.16 (−0.09 to 0.22)
**Number of other uploads**					
	Pedometer	0.99 (0.99-1.00)	0.99 (0.98-1.00)	0.11 (−0.05 to 0.07)	0.03 (−0.01 to 0.07)
	Weight	1.00 (0.99-1.01)	0.99 (0.98-1.01)	0.03 (−0.05 to 0.11)	0.02 (−0.01 to 0.05)
	Stress	1.00 (0.99-1.01)	0.998 (0.99-1.00)	0.001 (−0.04 to 0.04)	−0.005 (−0.02 to 0.01)
	Medication	1.00 (1.00-1.01)	1.00 (0.99-1.01)	−0.01 (−0.05 to 0.04)	−0.01 (−0.03 to 0.01)
**Number of challenges**					
	Pedometer	0.88 (0.45-1.72)	1.38 (0.51-3.77)	−2.39 (−7.49 to 2.72)	−0.68 (−3.20 to 1.85)
	Recipe	1.33 (0.31-5.81)	1.13 (0.13-9.68)	−1.89 (−12.93 to 9.15)	1.80 (−3.64 to 7.24)
**Optional events**					
	Web-based learning	1.26 (0.34-4.69)	0.54 (0.13-2.35)	3.64 (−6.17 to 13.44)	3.20 (−1.61 to 8.02)
	Cooking classes	1.01 (0.40-2.55)	0.73 (0.22-2.35)	−0.25 (−7.36 to 6.86)	2.21 (−1.28 to 5.70)
**Total number of intervention contacts**		1.03 (0.99-1.07)	1.06 (0.99-1.13)	−0.42 (−0.71 to −0.14)^h^	−0.18 (−0.32 to −0.04)^h^
	Behavioral contacts	1.07 (0.98-1.17)	1.05 (0.92-1.20)	−0.73 (−1.37 to −0.10)^i^	−0.14 (−0.45 to 0.18)
	Pharmaceutical contacts	1.01 (0.90-1.12)	1.06 (0.89-1.26)	−0.68 (−1.49 to 0.13)	−0.40 (−0.80 to −0.001)^i^
	Laboratory contacts	1.22 (0.91-1.64)	1.21 (0.76-1.92)	−0.71 (−2.8 to 1.41)	−0.53 (−1.57 to 0.51)
	Technical contacts	1.09 (0.91-1.31)	1.28 (0.91-1.82)	−0.50 (−1.84 to 0.84)	−0.25 (−0.91 to 0.41)
Total number of patient-initiated intervention contacts		1.07 (0.98-1.18)	1.16 (0.98-1.37)	−0.98 (−1.62 to −0.34)^h^	−0.34 (−0.66 to −0.02)^i^

^a^Office blood pressure goals are <140 mm Hg for systolic and <90 mm Hg for diastolic.

^b^OR: odds ratio.

^c^BP: blood pressure.

^d^Reduction in systolic BP ≥5 mm Hg or reduction in diastolic BP ≥3 mm Hg.

^e^SBP: systolic blood pressure.

^f^6-month office BP—baseline office BP.

^g^DBP: diastolic blood pressure.

^h^*P*<.01.

^i^*P*<.05.

**Figure 3:**
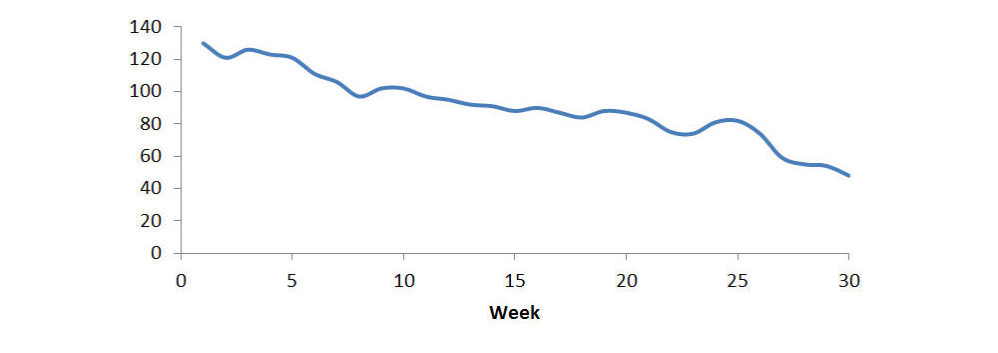
The number of participants meeting weekly home blood pressure monitoring frequency target (upload twice a day and 3 days in a week).

The number of participants meeting home BP monitoring frequency target (uploading twice a day and 3 days in a week) gradually decreased during the course of the intervention (Figure 3). Table 5 shows that more weeks of meeting home BP upload frequency target was significantly associated with higher odds of achieving home BP goals (*P*=.02); however, a higher number of total intervention, behavioral, pharmaceutical contacts were significantly associated with lower odds of achieving home BP goals (all *P*<.001). None of the intervention engagement measures was associated with improvements in values of home BP.

Additionally, none of the office and home BP outcomes were associated with baseline and change in participant activation, as measured by the PAM score (data not shown).

**Table 5:** Bivariate associations of home-monitored blood pressure (BP) with number of home BP, pedometer, weight, stress, and medication uploads and number of intervention contacts.

Intervention engagement measures		Achieving BP^a,b^ goals^c^ (n=133), OR^d^ (95% CI)	SBP^e^ change^f^ (n=132), coefficient (95% CI)	DBP^g^ change^f^ (n=132), coefficient (95% CI)
Number of weeks meeting home BP monitoring frequency target		1.05 (1.01-1.10)^h^	−0.09 (−0.33 to 0.15)	−0.07 (−0.21 to 0.07)
**Number of other uploads**				
	Pedometer	1.00 (1.00-1.01)	−0.02 (−0.07 to 0.03)	−0.01 (−0.03 to 0.02)
	Weight	1.00 (0.99-1.01)	−0.06 (−0.121 to 0.00)	−0.003 (−0.04 to 0.03)
	Stress	1.01 (1.00-1.02)	−0.01 (−0.04 to 0.02)	−0.01 (−0.02 to 0.01)
	Medication	1.01 (1.00-1.01)	−0.01 (−0.04 to 0.02)	−0.004 (−0.02 to 0.01)
**Number of challenges**				
	Pedometer	1.07 (0.50-2.30)	−0.80 (−4.81 to 3.21)	−0.38 (−2.66 to 1.90)
	Recipe	1.21 (0.23-6.30)	−6.19 (−14.43 to 2.05)	−0.62 (−5.34 to 4.10)
**Optional events**				
	Web-based learning	0.29 (0.07-1.17)	6.19 (−1.11 to 13.50)	3.78 (−0.36 to 7.93)
	Cooking classes	0.52 (0.21-1.30)	2.21 (−1.28 to 5.70)	1.03 (−1.89 to 3.95)
**Total number of intervention contacts**		0.90 (0.85-0.94)^i^	−0.08 (−0.31 to 0.15)	−0.07 (−0.20 to 0.06)
	Behavioral contacts	0.85 (0.76-0.94)^i^	−0.12 (−0.63 to 0.39)	−0.12 (−0.41 to 0.16)
	Pharmaceutical contacts	0.77 (0.67-0.89)^i^	−0.12 (−0.77 to 0.52)	−0.27 (−0.63 to 0.09)
	Laboratory contacts	0.81 (0.61-1.09)	−0.71 (−2.32 to 0.89)	−0.37 (−1.28 to 0.54)
	Technical contacts	1.06 (0.85-1.32)	−0.08 (−1.16 to 1.00)	0.06 (−0.55 to 0.68)
Total number of patient-initiated intervention contacts		0.95 (0.87-1.04)	−0.42 (−0.93 to 0.10)	−0.10 (−0.39 to 0.20)

^a^BP: blood pressure.

^b^Baseline home BP was an average of BP self-monitored during the 7 days after baseline, and 6-month home BP was an average of BP self-monitored during the 7 days before the 6-month visit.

^c^Home BP goals are <135 mm Hg for systolic and <85 mm Hg for diastolic.

^d^OR: odds ratio.

^e^SBP: systolic blood pressure.

^f^6-month home BP—baseline home BP.

^g^DBP: diastolic blood pressure.

^h^*P*<.05.

^i^*P*<.001.

## Discussion

### Principal Findings

This pre-post pilot study evaluated the EMPOWER-H program, a new personalized care delivery model utilizing an interactive Web-based disease management system integrated with the EHR for hypertension management. Compared with baseline, participants significantly reduced office-measured and home-monitored BP at 6 months, with 55.9% of the participants achieving office BP goals, 71.4% achieving home BP goals, and 86.0% achieving clinically meaningful reduction in office BP. The EMPOWER-H program also significantly decreased participants’ body weight; increased consumption frequency of fruit and vegetables, minutes of aerobic exercise, and knowledge; and reduced consumption frequency of high-salt and high-fat foods.

The findings of this study are consistent with previous studies showing that technology-assisted clinical tools and approaches hold great promise in improving the quality of hypertension management in the real world. The EMPOWER-H program led to a greater or similar reduction in systolic and diastolic office BP at 6 months compared with the amount of within-group reductions at 6 or 12 months achieved in previous information technology-supported interventions in primary care settings [34-39]. The EMPOWER-H program also resulted in similar percentage of patients meeting office BP goals compared with those achieving BP control in previous interventions [35,37].

The number of intervention contacts showed contrasting associations for office and home BP outcomes. Greater improvement in systolic or diastolic office BP was significantly associated with more intervention contacts, reflecting higher levels of clinical management. In contrast, higher odds of achieving home BP goals were significantly associated with fewer intervention contacts, reflecting lower levels of clinical management. We hypothesize that these results may be because of the clinical management challenges of different subsets of patients. Observation identified that some patients were highly motivated and able to meet their home BP goals independently, using their home BP data and adjustments in personal behavior. Other patients were less motivated, requiring significant clinical attention and more intervention contacts but achieving only moderate improvement in BP as manifested in office BP results. The contrasting results may also be partially explained by changes in the need for intervention contact over time, with more contacts needed in the beginning of the study and less contacts needed once some improvement was seen in home BP values.

### Population Health and Patient-Generated Health Data

Technology-assisted interventions in primary care are well aligned with a population health management strategy. About 90% of the American adults with poorly controlled hypertension have health insurance coverage and have received health care in the past year [40], suggesting an important opportunity to control hypertension in primary care settings. The US Preventive Services Task Force recommends that primary care providers offer or refer adults with CVD risk factors to intensive behavioral counseling interventions promoting a healthful diet and physical activity [41]. However, health care providers, including PCPs, nurse practitioners, and RNs usually do not have time or training to deliver lifestyle counseling themselves [42-44], suggesting that patient self-management is important for behavior change and disease management. In addition, the systems necessary to support self-management for larger groups of patient have not been readily available. Information technology-supported interventions in primary care, such as EMPOWER-H, have the potential to achieve hypertension control through facilitating patient engagement with support from health care providers. This study provides lessons for how such systems might be established effectively. The experience gained in this study provides support for the feasibility and value of using patient-generated health data, if properly structured and carefully collected, in the day-to-day clinical management of patients with chronic conditions. It allowed direct insights to clinical progress in the context of day-to-day life that were shared between patient and practitioner but without the need for the patient to visit the office. Reducing the need for office visits can potentially make such population health management strategies more efficient for patients, providers, and the health care system.

However, a number of barriers to wider use exist, and this study provides some insight into how these barriers may be overcome. In this study, whereas the primary outcome measure was office-measured BP, home-monitored values became the primary metric for day-to-day clinical management decisions. Using AHA recommended normal levels for home-monitored BP values provided clinical validity. The fact that patients could not alter the BP measurements allowed practitioners to build trust in the data over time. In addition, regular uploads showing patterns of BP changes, including insights to BP variability by time-of-day (eg, high BP only at night or only in the morning), presented new opportunities for personalized management. Although it is accepted that targets for home-monitored BP are lower than office-measured BP targets [20,45], there is limited real-world experience in the use of these lower targets for setting personal goals for home-monitored BP [46,47]. In addition, practicing clinicians are reported to value home-monitored BP less than office-measured BP for clinical management. They cite concerns about reliable monitors, patient instructions, and result interpretation [48], all of which are addressed by EMPOWER-H procedures.

The EMPOWER-H technology can be categorized as a population health software system. These systems are of increasing importance to health providers and payers to support the delivery of value-based care. A diverse range of information systems have been identified as supporting population health and subcategorization based on differentiation of features [49], and functional objectives can assist in understanding the primary aspect of population health management that individual systems are designed to support. Examples include: (1) systems that provide registry or analytic functions to assist the identification of populations with defined diagnoses and problems or facing specific risks, (2) systems that deliver evidence-based tasks known to drive improved outcomes and targeted to specific subpopulations, and (3) systems that drive the engagement of patients to comply with their personal program of care and make sustained changes to behaviors that positively impact their health outcomes. The EMPOWER-H system falls into this last category.

### Limitations

Our study has some methodological limitations such as small sample size, short intervention and follow-up duration, and lack of a control group. Furthermore, this study used a pre-post design, making it hard to discern whether the observed significant improvements were because of the EMPOWER-H intervention or the usual care treatments. Previous randomized controlled trials evaluated the primary care–based interventions that achieved less or similar amount of reduction in systolic and diastolic office BP compared with EMPOWER-H against a usual care condition [34-39]. The significant between-group differences favoring these interventions [34-39] suggest potential efficacy of EMPOWER-H compared with usual care. Despite these limitations, the study contributes to the current literature by testing an interactive Web-based disease management system linked to clinical workflows by interfacing self-monitoring devices (eg, BP monitor and pedometer), EHR, and a study dashboard for BP, weight, and lifestyle behavior management and communication between patients and providers. In addition, the retention rate of this study (99%) was higher than other similar 6-month studies [38,39,50], suggesting that the participants viewed the EMPOWER-H program as beneficial, and the format of the program delivery as acceptable.

Future studies are needed to answer the following questions: (1) What are the long-term effects of this kind of information technology–assisted interventions on clinical outcomes? (2) How could the approaches be further integrated into standard clinic workflows and the EHR, potentially relying more on patient-generated health data for routine care and reducing the need for office visits? (3) How could the approaches be automated (eg, an automated system for behavioral change, disease management, feedback, and risk notification for provider oversight), supporting patients to effectively self-manage their condition with lifestyle changes, to allow cost savings, and further transfer to the whole health care system at a scalable level? (4) How can these methods be applied to other clinical situations (eg, newly diagnosed hypertension and the management of other chronic diseases)? and (5) What is the cost-effectiveness and economic sustainability of such interventions when delivered at large scale?

### Conclusions

This study demonstrates that a Web-based system for BP management, with a focus on home BP monitoring driving personalized feedback and care-plan engagement, can be integrated with the EHR and can improve BP among adult patients with poorly controlled hypertension. Furthermore, this study provides insight to the feasibility and value of using patient-generated health data in the day-to-day management of chronic conditions. Questions about generalizability, scalability, and economic sustainability remain, and therefore, a large-scale pragmatic study with a longer follow-up period is warranted. If future studies show that these questions can be addressed, the program has the potential for widespread positive impact through implementation in primary care settings.

## Appendix

Multimedia Appendix 1 EMPOWER-H educational nugget examples.

Multimedia Appendix 2 Simplified diagram of EMPOWER-H population dashboard design.

Multimedia Appendix 3 Comprehensive tables of outcome measures used at baseline and 6 months.

## Abbreviations

AHA: American Heart Associatione
BMi: body mass index
BP: blood pressure
CVD: cardiovascular disease
DBP: diastolic blood pressure
EHR: electronic health record t
EMPOWER: Engaging and Motivating Patients Online With Enhanced Resources
EMPOWER-D: Engaging and Motivating Patients Online With Enhanced Resources-Diabetes
EMPOWER-H: Engaging and Motivating Patients Online With Enhanced Resources-Hypertension
HRQoL: heart-related quality of life
GED: general education diploma
NCM: nurse care manager
OR: odds ratio
PCP: primary care physician
PAM: Patient Activation Measure
PAMF: Palo Alto Medical Foundation
RD: registered dietitian
RN: registered nurse
SAS: Statistical Analysis System
SBP: systolic blood pressure
SD: standard deviation
VR-12: Veterans
RAND: 12-Item Health Survey
