# Supplementary material for: Personalized Hypertension Management Using Patient-Generated Health Data Integrated With Electronic Health Records (EMPOWER-H): Six-Month Pre-Post Study
Source: J Med Internet Res. 2017 Sep 19;19(9):e311. doi: 10.2196/jmir.7831 (PMC5627043; doi:10.2196/jmir.7831)
Supplement: Multimedia Appendix 1 [file jmir_v19i9e311_app1.pdf]

**Multimedia Appendix 1. EMPOWER-H educational nugget examples**. These were sent out to patients by their NCM via the EMPOWER system and could be received either on the phone app or by secure messaging within the patient portal to the EHR. Note: The linked content (i.e. a video, webpage, or PDF handout) was hosted on servers accessible from off the PAMF network.

| **Nugget Type** | **Title** | **Content** |
|---|---|---|
| Text with Link | DASH Diet | Have you heard of the DASH diet? It is an eating pattern that has been proven to lower blood pressure. To learn about the Dash Diet which stands for "Dietary Approaches to Stop Hypertension," click here for more information. |
| Text Only | Step Goal Achieved! | Congratulations on meeting your steps goal this past week! Keep up the good work. Remember that it can help to set slightly higher goals once you've achieved a certain level. Contact your Care Manager if you're ready to do that. Good work! |
| Video | Designing Goals | I am so glad to hear that achieving a healthy lifestyle is a high priority for you. I have included a valuable video about goal setting to help keep you on your path to a healthier life, please click here to view: [Video] Making a Healthy Lifestyle a Priority |
| Video | The Plate Method | The Plate Method is a simple way to learn how to eat healthy for lunch and dinner. Please take a look at this video to learn this very effective tool. Click here to learn more: [Video] The Plate Method |

Palo Alto Medical Foundation
A Sutter Health Affiliate

# Principles of the DASH Diet

## About the DASH diet

Research has shown that following a healthy eating plan can both reduce the risk of developing high blood pressure and lower an already-elevated blood pressure. For an overall eating plan, consider the DASH diet. "DASH" stands for "Dietary Approaches to Stop Hypertension," a clinical study that tested the effects of nutrients in food on blood pressure. Study results indicated that elevated blood pressures were reduced by an eating plan that emphasizes fruits, vegetables, and low-fat dairy foods as well as foods high in fiber and low in saturated fat, total fat and cholesterol. A second study showed that reducing the amount of sodium consumed lowers blood pressure. The DASH diet includes whole grains, poultry, fish, and nuts, and has reduced amounts of fats, red meats, sweets, sugar-containing beverages, and high-sodium processed foods.

# Guidelines for Managing Hypertension

## Reduce the sodium in your diet

- Choose fresh foods.
- Avoid adding salt at the table. Use herbs and spices liberally.
- Limit canned soups, frozen dinners and entrees and vegetables with sauces. If you must use these, get items labeled “low sodium.” You may rinse canned foods with water.
- Reduce your intake of bacon, sausage, hot dogs and luncheon meats such as corned beef, pastrami, bologna, ham, processed turkey and salami.
- Avoid smoked, pickled and cured foods.

## Achieve a healthy body weight

- Reduce your calories by choosing low-fat foods and eating smaller portions.
- Increase dietary fiber. Try to get 25 to 35 grams of fiber daily.
- Engage in regular exercise: at least 30 minutes of aerobic exercise daily to maintain weight and at least 60 minutes of aerobic exercise at least five times a week to lose weight.
- Practice modifying unhealthy eating behaviors.

## Increase foods that are high in potassium, magnesium and fiber

- Eat at least four to five servings a day of whole fresh fruit and four to five servings a day of vegetables. A serving of vegetable or fruit is approximately ½ cup cooked or one cup raw.
- Choose citrus fruit three times a week for potassium and fiber.

- Include nuts, seeds, dried beans and peas at least four times a week for potassium, magnesium and fiber.
- Eat tomatoes, oranges, bananas and potatoes for extra potassium.
- Choose whole grains and whole grain products for fiber and magnesium.

*Note – do not take potassium supplements unless instructed by your doctor, and those with kidney disease need to speak with their doctor before increasing potassium in their diet.

## Increase foods that are high in calcium

- Choose low-fat or fat-free cheese and yogurts and non-fat or 1% milk products daily.
- If you are lactose intolerant, use Lactaid or lactose-reduced milk.
- If you do not eat dairy products, supplement your diet with calcium supplement.

## Reduce caffeine

- Choose decaffeinated coffee, tea and diet sodas.
- If you do have caffeinated beverages, do not exceed two per day.
- Avoid caffeine-containing medications such as Anacin, Dristan, Excedrin Extra Strength, Midol, NoDoz and Vivarin.

## Drink adequate fluids

- A good rule of thumb is to drink eight cups of water per day.
- Fill up a pitcher of water and keep it on your desk or on the counter so that you are reminded to drink it.

## Quit smoking

- Reduce the numbers of cigarettes smoked.
- Seek assistance in quitting. Call 1-800-NOBUTTS.
- Discuss with your doctor.

## Take medication as directed by your doctor

- Do not skip your blood pressure medication.
- If you notice any side effects, notify your doctor.
- NSAIDS appear to boost blood pressure in older people with hypertension. They may also blunt the action of certain antihypertensive medications. Discuss the use of NSAIDs, such as Ibuprofen (Advil, Motrin), naproxen (Aleve) and ketoprofen (Orudis-KT) with your doctor.

## Following the DASH diet

The DASH eating plan shown below is based on 2,000 calories a day. The number of daily servings in a food group may vary from those listed depending on your caloric needs. Use this chart to help you plan your menus or take it with you when you go to the store.

| Food Group | Daily Servings | Serving Sizes | Examples and Notes | Significance of each food group to the plan |
|---|---|---|---|---|
| Grains and grain products | 6-8 | 1 slice bread<br>½-1 cup dry cereal<br>½ cup cooked rice, pasta, or cereal | whole wheat bread, English muffin, pita bread, bagel, cereals, grits, oatmeal | major sources of energy and fiber |
| Vegetables | 4-5 | 1 cup raw leafy vegetable<br>½ cup cooked vegetable<br>6 oz. vegetable juice | tomatoes, potatoes, peas, carrots, squash, broccoli, turnip greens, collards, kale, spinach, artichokes, beans, sweet potatoes | rich sources of potassium, magnesium and fiber |
| Fruits | 4-5 | 1 medium fruit<br>¼ cup dried fruit<br>½ cup fresh, frozen or canned fruit<br>1/2 cup fruit juice | apricots, bananas, dates, grapes, oranges, orange juice, grapefruit, grapefruit juice, melons, mangoes, peaches, pineapples, prunes, raisins, strawberries, tangerines | important sources of potassium, magnesium and fiber |
| Low-fat or fat-free dairy | 2-3 | 1 cup milk<br>1 cup yogurt<br>1½ oz. cheese | skim or 1% milk, skim or low-fat buttermilk, nonfat or low-fat yogurt, part skim mozzarella cheese, nonfat cheese | major sources of calcium and protein |
| Meats, poultry and fish | 6 or less | 1 oz. cooked meats, poultry or fish<br>1 egg or 2 egg whites | lean meats (trimmed of visible fat), broiled, roasted or baked; poultry with skin removed, limit egg yolk to four per week | rich sources of protein and magnesium |
| Nuts, seeds and legumes | 4-5 per week | 1½ oz. or 1/3 cup nuts<br>2 Tbsp. natural nut butter<br>½ oz. or 2 Tbsp. seeds<br>½ cup cooked legumes (dried beans or peas) | almonds, filberts, mixed nuts, peanuts, walnuts, sunflower seeds, kidney beans, lentils | rich sources of energy, magnesium, potassium, protein and fiber |

| | | | | |
|---|---|---|---|---|
| Fats and oils | 2-3 | 1 tsp. soft margarine, vegetable oil or regular mayonnaise<br>1 Tbsp. low-fat margarine or mayonnaise<br>2 Tbsp. light salad dressing | soft margarine, low-fat mayonnaise, light salad dressing, vegetable oil (such as olive, canola, corn or safflower) | besides fats added to foods, remember to choose foods that contain less fat |
| Sweets | 5 or less per week | 1 Tbsp. sugar<br>1 Tbsp. jelly or jam<br>½ ounce jelly beans<br>1 cup lemonade | maple syrup, sugar, jelly, jam, fruit-flavored gelatin, jelly beans, hard candy, fruit punch, sorbet, ices | sweets should be low in fat |

Source: The DASH Diet, NIH Publication No. 06-4082.

(Rev. 12/10)
