# Supplementary figures and images for: Personalized Hypertension Management Using Patient-Generated Health Data Integrated With Electronic Health Records (EMPOWER-H): Six-Month Pre-Post Study
Source: J Med Internet Res. 2017 Sep 19;19(9):e311. doi: 10.2196/jmir.7831 (PMC5627043; doi:10.2196/jmir.7831)

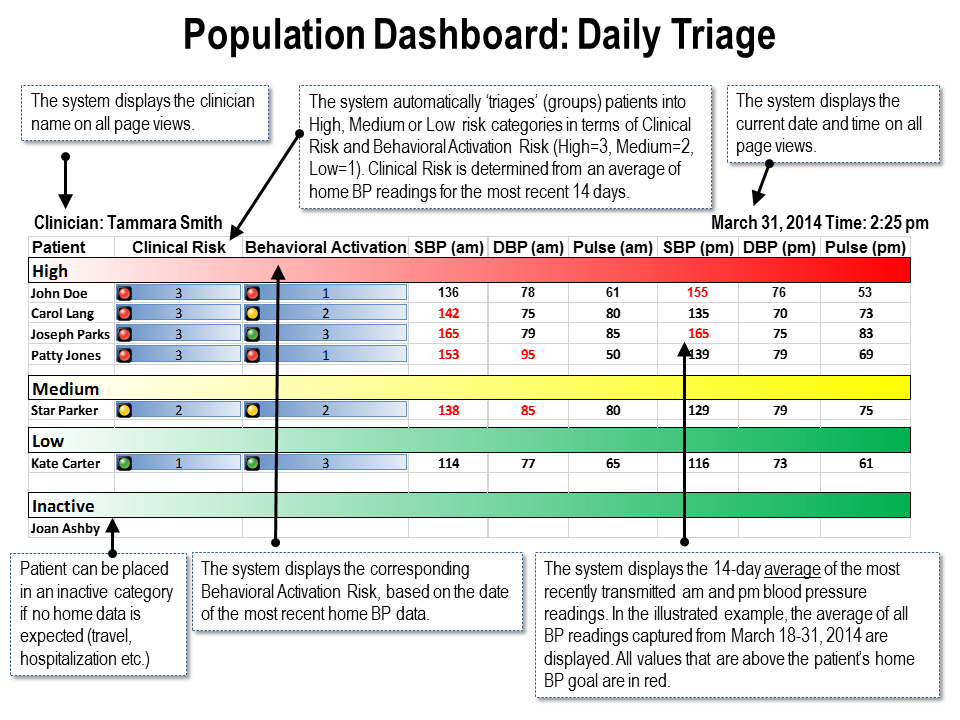

Supplement: Multimedia Appendix 2 [file jmir_v19i9e311_app2.png]
