# Supplementary material for: Personalized Hypertension Management Using Patient-Generated Health Data Integrated With Electronic Health Records (EMPOWER-H): Six-Month Pre-Post Study
Source: J Med Internet Res. 2017 Sep 19;19(9):e311. doi: 10.2196/jmir.7831 (PMC5627043; doi:10.2196/jmir.7831)
Supplement: Multimedia Appendix 3 [file jmir_v19i9e311_app3.pdf]

Multimedia Appendix 3. Comprehensive table of outcome measures used at baseline and 6 months.

**Measures**	**Source**	**Baseline**	**6 months**
**Primary Outcomes**			
Office-measured BP:<br>• Percent of participants achieving BP goals (<140/90 mmHg)	Biophysical	x	x
**Secondary Outcomes**			
Office-measured BP:<br>• Change in Systolic and Diastolic BP<br>• Percent of participants achieving clinically meaningful reductions in BP (a drop in Systolic BP of ≥5 mmHg or a drop in Diastolic BP of ≥3 mmHg)<br>Home-monitored BP<br>• Percent of participants achieving BP goals (<135/85 mmHg)<br>• Change in Systolic and Diastolic BP	Biophysical	x	x
Body weight	Biophysical	x	x
Intervention engagement (number and type of Care Team contacts, education messages, and education videos)	Intervention process measures	⟷	
BP Self-monitoring:<br>• Number of participants meeting weekly home BP monitoring frequency target (upload twice a day and three days in a week)	Automated home BP monitoring tools	⟷	
Other self-monitoring activities:<br>• Number of stress uploads<br>• Number of medication uploads<br>• Number of pedometer uploads<br>• Number of weight uploads<br>• Number of challenges (i.e., pedometer challenge, recipe challenge)<br>• Number of optional events (i.e., cooking classes, online learning webinars)	Online tools and smartphone application	⟷	
Patient activation (Patient Activation Measures)	Self-administered survey	x	x
Diet	Self-administered survey	x	x
Physical activity	Self-administered survey	x	x

	Smoking	Self-administered survey	x	x
	Hypertension knowledge	Self-administered survey	x	x
	Health-related quality of life (Veteran's Rank 12-item Health Survey)	Self-administered survey	x	x
**Potential effect modifiers**				
	Sociodemographics (age, sex, race)	Self-administered survey	x	
	Weight status (height, weight)	Biophysical	x	
	Patient Activation (Patient Activation Measures)	Self-administered survey	x	
